# Specific retinal neurons regulate context-dependent defensive responses to visual threat

**DOI:** 10.1093/pnasnexus/pgae423

**Published:** 2024-09-24

**Authors:** Tracy Lee, Hannah Weinberg-Wolf, Thomas E Zapadka, Andrii Rudenko, Jonathan B Demb, In-Jung Kim

**Affiliations:** Department of Ophthalmology and Visual Science, Yale University School of Medicine, New Haven, CT 06511, USA; Department of Ophthalmology and Visual Science, Yale University School of Medicine, New Haven, CT 06511, USA; Department of Cellular and Molecular Physiology, Yale University School of Medicine, New Haven, CT 06511, USA; Department of Biology, Graduate Programs in Biology and Biochemistry, City College and City University of New York, New York, NY 10031, USA; Department of Ophthalmology and Visual Science, Yale University School of Medicine, New Haven, CT 06511, USA; Department of Cellular and Molecular Physiology, Yale University School of Medicine, New Haven, CT 06511, USA; Department of Neuroscience, Yale University School of Medicine, New Haven, CT 06511, USA; Wu Tsai Institute, Yale University, New Haven, CT 06511, USA; Department of Ophthalmology and Visual Science, Yale University School of Medicine, New Haven, CT 06511, USA; Department of Neuroscience, Yale University School of Medicine, New Haven, CT 06511, USA; Wu Tsai Institute, Yale University, New Haven, CT 06511, USA

**Keywords:** defensive responses, visual threat, retinal ganglion cells, shelter, environmental context

## Abstract

While encountering a visual threat, an animal assesses multiple factors to choose an appropriate defensive strategy. For example, when a rodent detects a looming aerial predator, its behavioral response can be influenced by a specific environmental context, such as the availability of a shelter. Indeed, rodents typically escape from a looming stimulus when a shelter is present; otherwise, they typically freeze. Here we report that context-dependent behavioral responses can be initiated at the earliest stage of the visual system by distinct types of retinal ganglion cells (RGCs), the retina's output neurons. Using genetically defined cell ablation in mature mice, we discovered that some RGC types were necessary for either escaping (alpha RGCs) or freezing (intrinsically photosensitive RGCs) in response to a looming stimulus but not for both behaviors; whereas other RGC types were not required for either behavior (direction-selective RGCs preferring vertical motion). Altogether, our results suggest that specific RGC types regulate distinct behavioral responses elicited by the same threatening stimulus depending on contextual signals in the environment. These findings emphasize the unique contribution of early visual pathways to evolutionally conserved behavioral reactions.

**Significance Statement**

Depending on the environment, an animal will respond differently to a visually threatening looming stimulus: escaping if a shelter is present and otherwise freezing. The neuronal mechanisms underlying these two behaviors apparently depend on distinct circuits in the midbrain, but it is not known whether they likewise depend on distinct circuits earlier in the visual system. Here, we demonstrate that distinct types of retinal ganglion cell, the retina's output neurons, independently contribute to behavioral choice in response to the same looming stimulus, depending on the availability of a shelter. Our findings highlight the importance of information processing by specific retinal neurons in regulating complex behaviors.

## Introduction

Threatening visual inputs, such as approaching objects, trigger defensive behaviors in both humans and animals. Humans react to looming shadows or approaching objects with integrated avoidance responses (1, 2). Animals are threatened by an approaching aerial predator, which can be mimicked in the laboratory by a looming stimulus: an expanding dark spot on an overhead computer monitor. In response to looming, a mouse elicits one of two defensive responses: freezing or escaping (3). Freezing presumably makes the animal difficult to see, and escaping makes the animal difficult to capture. To enhance survival probability, animals select an appropriate behavior after integrating several relevant factors, including the proximity of the threatening stimulus, internal states, and environmental context (4–6). Experiments that altered neuronal activity in specific cell types demonstrated that distinct brain circuits, mainly involving the superior colliculus (SC), separately regulate freezing and escaping behaviors (7–11). These results suggest that distinct brain circuits control different behaviors to the same stimulus, but it remains unclear how early in the visual system distinct cell types mediate alternative behavioral choices.

Visual information processing begins in the retina, where the extracted signals are conveyed by retinal ganglion cells (RGCs) to downstream brain areas, including the SC. Mice have >40 types of RGCs, many of which would presumably respond to the dark moving edge of the looming stimulus, either with an increase (OFF cells) or decrease (ON cells) in firing (12–16). Several RGC types are apparently necessary for the defensive response to looming, because the behavior is impaired if the RGC types are ablated (17–20). Indeed, manipulation of either individual RGC types or groups of types impacted both freezing and escaping reactions, which were typically tested individually across studies. However, we do not understand whether certain RGC types can differentially influence the behavioral choice to looming depending on environmental context. In one study, using multiple stimulus paradigms, a broad group of RGC types defined by expression of the transcription factor Brn3b (Brn3b+) were necessary for escaping behavior to a looming stimulus but not for freezing behavior to another lateral-motion (sweeping) stimulus (21). Furthermore, the interpretation of this phenotype is complicated by the deletion of Brn3b + RGCs during development and its potential impact on circuit assembly.

Here we tested whether selection of a behavioral response to the looming stimulus can be initiated by specific RGC types depending on the environmental context, i.e. the presence versus absence of a shelter. To selectively target individual RGC types, we utilized well-characterized mouse lines in which distinct RGC types are marked by expression of Cre-recombinase. To ablate RGC types in each transgenic line, we made intraocular injections of a genetically encoded toxin with Cre-dependent expression in adult mice and examined defensive responses either with a shelter (to probe escaping) or without a shelter (to probe freezing). Our results show that alpha RGCs are necessary for the escaping response only; intrinsically photosensitive RGCs (ipRGCs) are necessary for the freezing response only; and vertical motion-preferring ON–OFF direction-selective RGCs (vertical ooDS-RGCs) are not necessary for either behavior. Our results show that distinct RGC types can regulate defensive strategies to the same threatening visual stimulus in a context-dependent manner. This finding emphasizes the importance of information processing at the retinal level for complex behaviors.

## Results

### Using the looming stimulus assay to test defensive behavior following RGC ablation

**Fig. 1.**
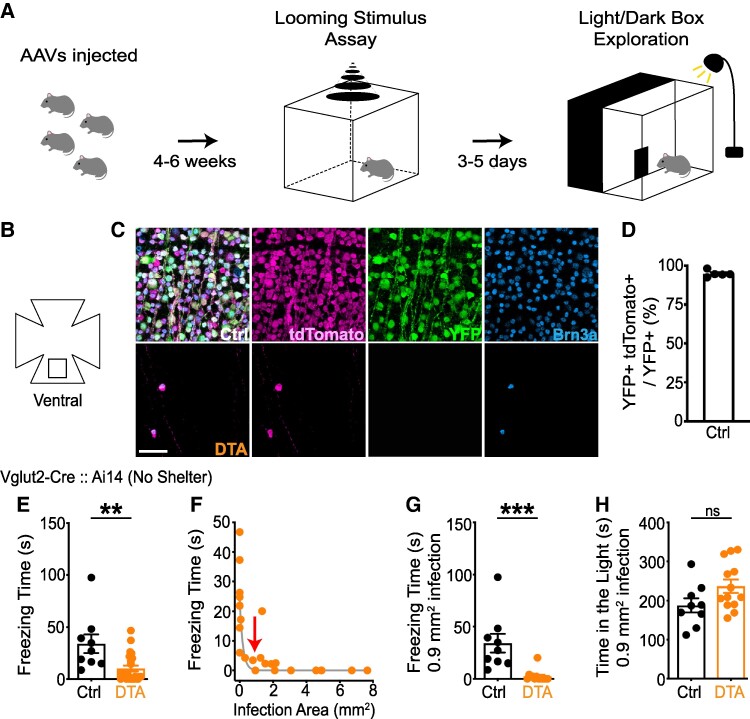
Assessment of the ablated retinal area minimally required to trigger looming-evoked behavior. A) Schematic diagram of the behavioral paradigms. The looming stimulus was presented on the ceiling of a chamber, either with or without a shelter. 3–5 days later, the mice were subjected to the light/dark exploration test. B–D) Schematic diagram showing the area of ventral retina imaged for histological analysis (boxed; (B)). Either AAV-FLEX-YFP or AAV-FLEX-DTA (C) was injected into both eyes of Vglut2-Cre:: Ai14 mice. Following the behavioral assays, retinas were dissected and immunostained against DsRed (recognizing tdTomato; magenta), YFP (green), S-opsin (marking ventral retina) and Brn3a (a pan RGC marker; cyan). The Brn3a antibody provided reliable staining of RGCs and was compatible with quadruple immunostaining protocols. Most (∼95.2%) YFP + cells were tdTomato+ (i.e. Vglut2+) in controls, validating the FLEX strategy. Very few, if any, Brn3a + RGCs were detected in the ventral retina of DTA-injected mice (D). E–G) RGC ablation significantly decreased the total freezing time, with an exponential relationship [fitted line: *y* = 24.2exp(-*x*/0.185)]. A 0.9 mm^2^ (arrow) ventral area infection strongly reduced freezing responses. H) The time in the light on the light/dark exploration test was similar in control and DTA-infected mice. Unpaired two-tailed Student's t-test (mean ± SEM, *P* = 0.002 for (E); *P* < 0.001 for (G); *P* = 0.067 for (H). Scale bars: 50 µm. See also Tables S1 and S2.

We started by performing a positive control experiment to validate our cell ablation method and to test the impact of cell ablation on a robust visual behavior. For this purpose, we targeted all types of RGC, using the Vglut2-Cre line (16) crossed to a Cre-dependent reporter line (td-Tomato; Ai14), and we used our standard looming stimulus assay without a shelter, because it robustly elicits a freezing response (22). To ablate RGCs, we delivered adeno-associated virus (AAV) expressing Cre-dependent Diphtheria Toxin A subunit (FLEX-DTA) into both eyes. Control mice instead received an AAV expressing Cre-dependent yellow fluorescent protein (AAV-FLEX-YFP). We targeted the AAV injection to the ventral area of the retina, where the looming stimulus projects (see Methods). Four to six weeks later, mice were evaluated in the looming stimulus assay without a shelter to examine freezing responses (Fig. 1A). Three to five days later, a light/dark exploration test was conducted to assess basic visual abilities. To analyze the AAV-infected area, retinas were dissected and immunostained against td-Tomato (i.e. all RGCs), YFP (i.e. AAV expression) and Brn3a [i.e. most RGCs (23)]. We also included an antibody to S-opsin, which marks the high density of S-cones (short-wavelength sensitive) in the ventral retina (24–26) (Fig. 1B–D). In control mice, ∼95.2% of YFP + cells were td-Tomato+, confirming the fidelity of the FLEX system (Table S1).

After the onset of the looming stimulus, DTA-injected mice froze significantly less than controls, confirming the requirement of RGCs for this visual behavior (Fig. 1E–H). The area of AAV infection (i.e. the area of RGC ablation) required to reliably abolish freezing was >0.9 mm^2^ of ventral retina (with >90% cell ablation within this area), which served as a guide for experiments with the other Cre lines (see Methods for selection criteria). Further, DTA-injected Vglut2-Cre mice behaved similarly to controls in the light/dark exploration test, although there was a trend for DTA-injected mice to spend relatively more time in the brightly lit area. This observation suggested that RGCs outside the ventral retina can mediate this simple light-avoidance behavior (Fig. S1).

For the Cre lines used below, with selective RGC expression, we tested looming-evoked behavior both with and without a shelter. The goal was to link specific RGC types to specific behaviors that depend on environmental context. In the presence of a shelter, some mice exhibited momentary freezing prior to escaping, and others froze instead of escaping. To capture the behavioral phenotypes with a shelter, the following parameters were quantified: ratio of mice entering the shelter; initial freezing time; escape latency; and average speed before and after stimulus onset. To avoid habituation, separate cohorts were utilized in experiments with and without a shelter, and each mouse was tested on the looming assay only once.

### Alpha RGCs are necessary for escaping responses only

**Fig. 2.**
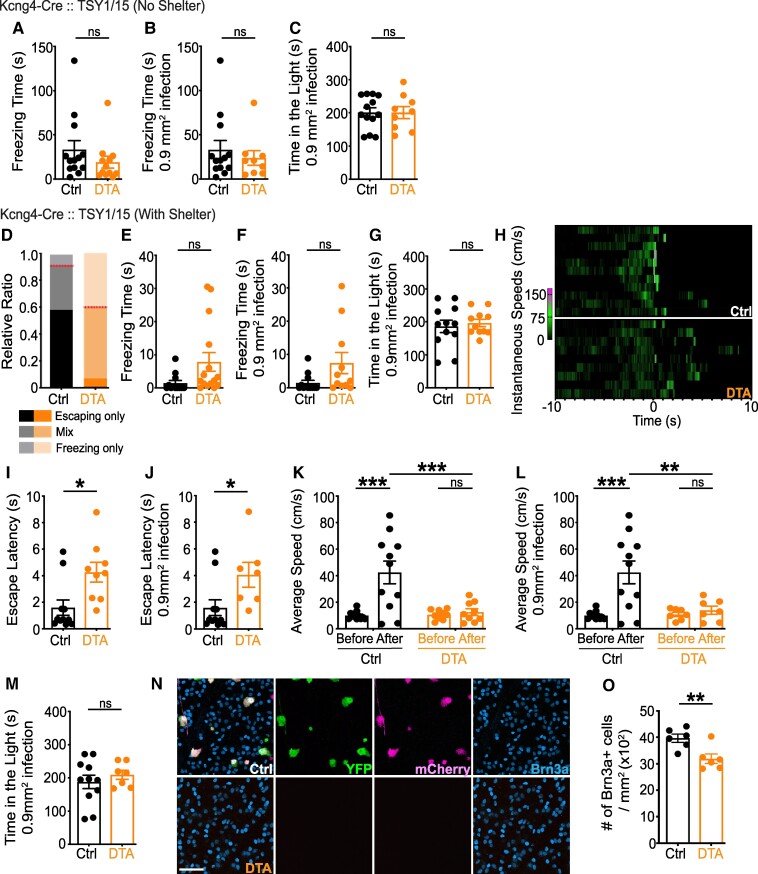
Alpha RGCs are necessary only for the escaping response to looming. Either AAV-FLEX-mCherry or AAV-FLEX-DTA was injected into both eyes of Kcng4-Cre:: TSY1 (or TSY15) mice expressing Cre-dependent YFP (55). A–C) Without a shelter, ablation of alpha RGCs had no effect on freezing time (A and B). Control and ablated groups behaved similarly in the light/dark exploration test (C). D–M) With a shelter, ∼40% of DTA-infected mice did not enter the shelter and momentarily froze (D), resulting in a trend of increased freezing time, compared to controls (E and F). Red lines (D) indicate the ratio of mice that entered the shelter (*n* = 11/12 for control, *n* = 9/15 for ablation). Heat maps show instantaneous speed 10 s before and after stimulus onset of individual mice that entered the shelter (H). Ablation of alpha RGCs increased escape latency (I and J). The looming stimulus increased speed of movement in control but not in ablated group. A significant difference in post-stimulus speed was observed between control and ablated group (K and L). No group difference was found in the light/dark exploration test (G and M). N–O) Following behavioral assays, retinas were collected and immunostained against GFP (green), DsRed (magenta), S-opsin, and Brn3a (a pan RGC marker; cyan). Approximately 19.4% of Brn3a + RGCs were ablated in the ventral retina of DTA-injected mice, confirming a selective ablation. Unpaired two-tailed Student's t-test (mean ± SEM, *P* = 0.260 for (A); *P* = 0.512 for (B); *P* = 0.994 for (C); *P* = 0.059 for (E); *P* = 0.070 for (F); *P* = 0.387 for (G); *P* = 0.010 for (I); *P* = 0.031 for (J); *P* = 0.448 for (M); *P* = 0.009 for (O)). Tukey post-hoc analysis (mean ± SEM, control, before and after stimulus: *P* < 0.001 for (K and L); ablated mice, before and after stimulus: *P* = 0.993 for (K), *P* = 0.984 for (L); control and ablated mice, after stimulus: *P* < 0.001 for (K), *P* = 0.006 for (L)). Scale bars: 50 µm. See also Tables S1 and S2.

Alpha RGCs comprise four types: ON and OFF Transient and ON and OFF Sustained (27). The OFF Transient alpha RGC was a primary candidate for encoding the looming stimulus given its strong firing to expanding dark stimuli (19, 28). To manipulate alpha RGCs, we used the Kcng4-Cre line, which labels four alpha RGC types (27, 29). Unexpectedly, ablation of alpha RGCs did not significantly affect freezing in the absence of a shelter (Fig. 2A and B). With a shelter, however, more DTA-injected mice (∼40%) than controls (∼8%) did not enter the shelter and instead froze (Fig. 2D). Further, we observed a trend towards increased momentary freezing time in DTA-injected mice (Fig. 2E and F). Among those entering the shelter, controls exhibited a relatively shorter latency (Fig. 2I and J) and increased speed following stimulus onset (Fig. 2K and L). A 2 × 2 ANOVA (ablation × looming stimulus) showed main effects of ablation (*F* = 8.753, *P* = 0.005) and looming stimulus (*F* = 12.10, *P* = 0.001) with a strong interaction (*F* = 9.454, *P* = 0.004). The same was true for the subset of animals with >0.9 mm^2^ area of Cre + cells ablated in the ventral retina, with main effects of ablation (*F* = 6.068, *P* = 0.019) and looming stimulus (*F* = 9.994, *P* = 0.003) with a significant interaction (*F* = 6.786, *P* = 0.014). Follow-up post-hoc analysis revealed that controls moved at faster speeds than DTA-injected mice after stimulus onset. No difference was observed between groups during the light/dark exploration test (Fig. 2C, G and M).

For histological analysis, retinas ­were dissected and immunostained against YFP (alpha RGCs), mCherry (AAV), Brn3a (most RGCs), and S-opsin (ventral retina; Fig. 2N, O and Fig. S1). Approximately 19.4% of Brn3a + RGCs were ablated in the ventral retina of DTA-injected mice, consistent with a selective deletion of alpha RGCs. Given that the Kcng4-Cre line labels some interneurons (30), in addition to alpha RGCs, we examined cells labeled in the inner nuclear layer (INL) and found that only ∼1.5% of YFP + cells were mCherry+ (Fig. S2 and Table S1), verifying a selective labeling of RGCs by the AAVs used here. These data rule out nonspecificity of cell ablation following intraocular injections and demonstrate our ability to precisely manipulate and identify RGC types that mediate defensive responses. Together, our findings suggest that ablation of alpha RGCs specifically affects escaping responses to the looming stimulus. Our finding of impaired escaping behavior in the presence of a shelter supports a similar impairment observed after deleting OFF Transient alpha RGCs more specifically, using a different Cre line (19), which is described further in Discussion.

### Vertical ooDS-RGCs are not necessary for either freezing or escaping responses

**Fig. 3.**
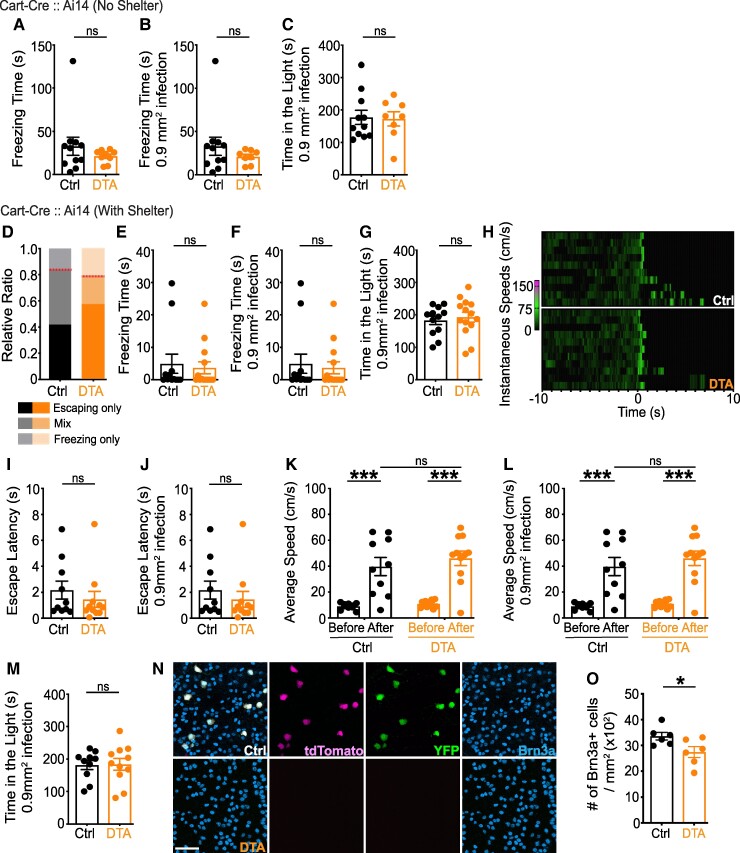
Vertical ooDS-RGCs are not necessary for either freezing or escaping responses. Either AAV-FLEX-YFP or AAV-FLEX-DTA was injected into both eyes of Cart-Cre:: Ai14 mice. A–C) Without a shelter, ablation of vertical ooDS-RGCs had no effect on freezing time even after considering the infection area (A and B). No difference was observed in the light/dark exploration test (C). D–M) With a shelter, most mice in both groups entered the shelter (D) with no difference between groups in momentary freezing time (E and F). Red lines (D) indicate the ratio of mice that entered the shelter (*n* = 10/12 for control, *n* = 11/14 for ablation). Heat maps show instantaneous speed 10 s before and after stimulus onset of individual mice that entered the shelter (H). No difference was detected in escape latency (I and J). Looming stimulus increased speed of movement in both control and DTA-infected mice; however, no significant difference after stimulus was detected between groups (K and L). No group difference was found in the light/dark exploration test (G and M). N–O) For post-hoc histological analysis, retinas were immunostained against GFP (green), DsRed (magenta), S-opsin and Brn3a (Cyan). Approximately 18.4% of Brn3a + RGCs were ablated in the ventral retina of DTA-injected mice, validating a selective ablation. Unpaired two-tailed Student's t-test (mean ± SEM, *P* = 0.348 for (A); *P* = 0.354 for (B); *P* = 0.879 for (C); *P* = 0.714 for (E and F); *P* = 0.635 for (G); *P* = 0.448 for (I and J); *P* = 0.925 for (M); *P* = 0.038 for (O)). Tukey post-hoc analysis (mean ± SEM, *P* < 0.001 for control before and after stimulus; *P* < 0.001 for ablated mice before and after stimulus; *P* = 0.747 for control and ablated mice after stimulus for (K and L)). Scale bars: 50 µm. See also Tables S1 and S2.

The ooDS-RGCs detect the direction of moving stimuli, primarily in each of four directions (two vertical, two horizontal), and transmit this information to central targets, including the midbrain (31–33). To ablate ooDS-RGCs, we used the Cart-Cre line crossed to the Ai14 line. The Cart-Cre line labels two populations of ooDS-RGCs sensitive to vertical (i.e. up or down) motion (vertical ooDS-RGCs) (34); consistent with this report, our immunostaining showed that ∼42.0% of Cart + RGCs are td-Tomato+ (Fig. S2 and Table S1). The looming stimulus assay showed that without a shelter, ablation of vertical ooDS-RGCs had no effect on freezing time (Fig. 3A and B). With a shelter, most animals (79–83%) in both control and DTA-infected groups entered the shelter (Fig. 3D–L). No between-group differences were identified in any measured behavioral parameter. Likewise, no difference was detected in the light/dark exploration test (Fig. 3C, G and M). The post-hoc histological analysis revealed that ∼18.4% of RGCs were ablated in ventral retina (Fig. 3N, O and Fig. S1), confirming a selective manipulation of RGCs. We also found that ∼13.5% of td-Tomato + neurons in the INL were YFP+ (Fig. S2 and Table S1), suggesting that some interneurons might be ablated by the AAV strategy, but, in any case, this did not result in a measurable phenotype. Together, these results suggest that vertical ooDS-RGCs are not required for any defensive responses to the looming stimulus.

### ipRGCs are necessary for freezing responses only

We utilized the Opn4-Cre line to target melanopsin-expressing ipRGCs, which includes six cell types (M1–M6) (35–38). Each ipRGC type combines a slow intrinsic photoresponse with fast synaptic input. One of these types, the M4, is the ON Sustained alpha RGC (35, 39), also labeled in the Kcng4-Cre line above. All ipRGCs are ON RGCs (35, 40, 41), whereas the dark looming stimulus seems best encoded by OFF RGCs (28). Thus, we expected ipRGCs to have little influence on looming-evoked responses.

**Fig. 4.**
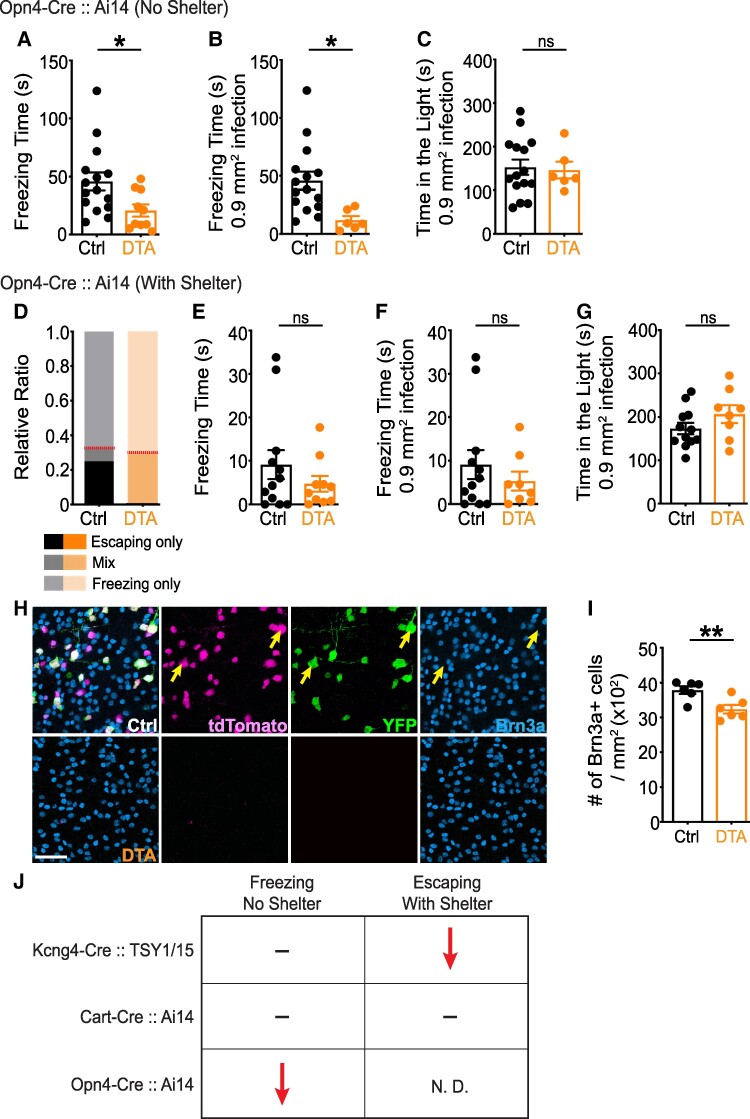
ipRGCs are necessary only for the freezing response to looming. Either AAV-FLEX-YFP or AAV-FLEX-DTA was injected into both eyes of Opn4-Cre:: Ai14 mice. A–C) Without a shelter, ablation of ipRGCs decreased freezing time (A and B). No difference was found in the light/dark exploration test (C). D–G) With a shelter, most mice in both groups did not enter the shelter and instead froze (D). Red lines (D) indicate the ratio of mice that entered the shelter (*n* = 4/12 for control, *n* = 3/10 for ablation). No significant difference was observed in the momentary freezing responses (E and F). No group difference was found in the light/dark exploration test (G). H–I) Following behavioral analysis, retinas were immunostained against GFP (green), DsRed (magenta), S-opsin and Brn3a (cyan). Approximately 14.5% of Brn3a + RGCs were ablated in the ventral retina of DTA-injected mice, validating a selective ablation. Yellow arrows indicate M4 RGCs based on soma size and weak (or absent) Brn3a labeling. Unpaired two-tailed Student's t-test (mean ± SEM, *P* = 0.025 for (A); *P* = 0.015 for (B); *P* = 0.835 for (C); *P* = 0.288 for (E); *P* = 0.408 for (F); *P* = 0.169 for (G); *P* = 0.008 for (I)). Scale bars: 50 µm. See also Tables S1 and S2. J) Summary of defensive responses to the looming stimulus following ablation of specific RGC types in each transgenic line. N.D., not determined: the conclusion could not be determined because of the very limited number of mice that entered the shelter in both control and ablation conditions.

Unexpectedly, without a shelter, ablation of ipRGCs decreased freezing responses significantly compared to controls (Fig. 4A and B). Further, with a shelter, most control and DTA-injected mice (66–70%) did not enter the shelter and instead temporarily froze, with similar freezing times between the groups (Fig. 4D–F). Other parameters of escaping responses were not analyzed due to the small number of animals that escaped in either group. The low escaping ratio of Opn4-Cre control mice, compared to other transgenic lines, is presumably related to the genetic background of this line (see Discussion). No group differences were found in the light/dark exploration test (Fig. 4C and G). Following behavioral experiments, we immunostained retinas and found that ∼14.5% of Brn3a + RGCs were ablated, consistent with the fraction of RGCs labeled in the Opn4-Cre line (Fig. 4H, I and Fig. S1) (42). Quantification of M4 RGCs, based on their large soma size and weak (or absent) Brn3a labeling (27), showed that ∼90% were ablated in the DTA-infected ventral retina, suggesting that M4 RGCs were not preferentially spared (Fig. 4H and Table S1). Analysis in the INL and outer nuclear layer revealed that <1% of td-Tomato + neurons were YFP+ (Fig. S2 and Table S1), suggesting that our manipulation did not affect neurons outside the ganglion cell layer. Overall, we found that ablation of ipRGCs specifically impaired freezing responses to the looming stimulus in the absence of a shelter; and the Opn4-Cre:: Ai14 genetic background unexpectedly suppressed escaping responses in the presence of a shelter.

## Discussion

Our main finding is that ablation of distinct RGC types (i.e. those expressing *Kcng4* versus *Opn4*) can differentially impair two behaviors (i.e. escaping versus freezing) to the same visual stimulus (looming), depending on the availability of a shelter. This suggests that distinct retinal circuits differentially contribute to complex and specific behavioral repertoires to the same visual stimulus based on the environmental context (i.e. presence versus absence of a shelter).

Following alpha RGC ablation, we found a deficit in escaping to a shelter, which is consistent with impaired escape behavior after ablating OFF Transient alpha cells more selectively using the Kcnip2-CreER line (19). However, without a shelter, we observed no effect on freezing behavior after ablating alpha RGCs; whereas Wang et al. (19) reported decreased freezing after manipulating OFF Transient alpha cells with an inhibitory optogenetic method (activation of ArchT). This discrepancy could possibly be explained by the different strategies for manipulating RGC activity. Further, given that the Kcnip2-CreER line also labeled cells in the INL, it is possible that the decreased freezing in the optogenetic experiment could be explained by effects on interneurons. Another difference between the two studies is mouse age. While Wang et al. utilized ∼2-month-old (young adult) mice, we studied 4–5-month-old (mature) animals that have fully developed brain sub-systems, including olfactory and limbic structures (43), which can modulate a variety of behaviors. Further, the Kcng4-Cre line we employed labels three alpha RGC types in addition to the OFF Transient alpha RGCs labeled in the Kcnip2-CreER line, which could alter the balance of impaired pathways; although it would be surprising if this made the behavioral phenotype more restrictive in the current study. Overall, our results showed that ablation of alpha RGCs differentiate context-dependent behavioral responses to an identical looming stimulus in two environmental contexts (presence versus absence of a shelter) using natural visual input.

In contrast to the Kcng4-Cre line, ablating RGCs in the Opn4-Cre line impaired the freezing response to the looming stimulus in the absence of a shelter. This raises the question, which ipRGCs contribute to the freezing response? The M4 ipRGC (i.e. ON sustained alpha RGC) is targeted in both the Kcng4- and Opn4-Cre lines, suggesting that ablation of these RGCs would not explain the differential behavioral effects in the two lines. The other ipRGC types (M1-3, M5-6) collectively project to brain areas involved in defensive behaviors to visual threat (ventral lateral geniculate nucleus and SC) (5, 8, 44). Interestingly, these ipRGCs are all ON cells, but they could respond to the expanding dark spot by decreasing their baseline firing rates (45). Determining the roles of specific ipRGC types in freezing behavior will require further studies.

Notably, mice with the Opn4-Cre:: Ai14 genetic background, in both control and DTA groups, primarily exhibited a freezing response to the looming stimulus, regardless of whether a shelter was present. Interestingly, a similar phenotype was reported in one species of deer mice, which naturally inhabit open fields; this contrasts with the deer mice's sister species that reside in the underbrush (46). This behavioral difference was mediated by differential information processing in the midbrain, apparently linked to alterations of the relevant neural circuitry as an evolutionary adaptation to the distinct living conditions. Here, we did not determine the mechanistic basis of unusual freezing responses by Opn4-Cre:: Ai14 mice. In the future, it could be informative to determine whether the phenotype in these mice depends on the loss of one copy of the Opn4 allele, due to Cre insertion, or, alternatively, whether the mixed genetic backgrounds of Opn4-Cre:: Ai14 lines caused structural and/or functional alterations of neural circuitry linked to threat-evoked behavior.

We analyzed the impact of deleting ooDS-RGCs because their DS responses would apparently encode the moving edges of the looming stimulus. The DS response of the ooDS-RGCs is known to be mediated by presynaptic input from ON and OFF starburst amacrine cells (SACs) (33). Interestingly, two previous studies on behavior to the looming stimulus that ablated SACs reported conflicting results: Wang et al. (19) reported that ablation of SACs had no effect on the escaping response to the looming stimulus, whereas Bohl et al. (47) reported that ablation of OFF SACs specifically abolished defensive responses. We expected that direct manipulation of ooDS-RGCs would reveal the importance of DS mechanisms in the looming response. However, considering that the Cart-Cre line labels vertically tuned ooDS-RGCs only (34), our conclusion that ooDS-RGCs are not critical for freezing or escaping response is limited to these populations of ooDS-RGCs. Future experiments using a mouse line targeting the complete complement of ooDS-RGCs could resolve the role of ooDS-RGCs in visually triggered defensive responses.

The light/dark exploration test has been used to examine visual restoration in blind mice (48–50). Mice tend to avoid a brightly lit area, providing a simple assessment of basic vision. The same behavioral paradigm has been employed to test anxiety-like features based on two conflicting mouse instincts: the urge to explore a novel environment and the urge to avoid an illuminated area. The second urge dominates in an anxious mouse, which avoids the light. Activating ipRGCs, either with natural light or by chemogenetics, increases anxiety-related behavior (51–53), and stress-induced anxiety can enhance the response to looming (54). Therefore, manipulating ipRGCs’ activity in our experiments may have influenced defensive responses to the looming threat indirectly, by influencing the level of anxiety. However, the observed behavioral similarity between controls and DTA-injected mice in the light/dark exploration tests, strongly suggests that anxiety was not altered in the experimental animals and likewise did not contribute to the phenotypes in the looming stimulus assay.

Using our cell ablation method, it would be possible to evaluate the relationship between RGC types and other behaviors. For example, defensive behavior can be induced by an overhead sweeping stimulus (i.e. laterally moving overhead spot), which evokes a freezing response (4). It would be interesting to test whether this behavior depends on the cell types studied here. However, given the deletion of RGCs of interest within a confined retinal area (e.g. ventral retina), our method would be less useful for visual behaviors that likely depend on input across the entire retina (e.g. pupillary light reflex).

Strengths of our study include the focus on ablating RGCs rather than interneurons, which influence multiple RGC types (15, 47). Also, we utilized a different cohort of mice in each experiment (control versus ablation) to ensure that mice would not habituate to the stimulus. Lastly, compared to previous studies that investigated the role of RGCs in looming-evoked behavior (17–21), ours is unique in comparing freezing and escaping responses to the same looming stimulus in mature (>4 month old) mice after ablating genetically defined RGC types; and our approach examined different mouse lines in identical housing conditions. In conclusion, we identified distinct RGC types selectively mediating freezing and escaping responses to visual threat independently (Fig. 4J), emphasizing the importance of information processing by specific retinal circuits in regulating complex survival-related behavior.

## Materials and methods

### Mice

All animal procedures were approved by the Institutional Animal Care and Use Committee at Yale University. Mice were maintained in pathogen-free facilities at Yale University under standard housing conditions with food and water *ad libitum*. Intraocular injection was performed on adult mice (12–16 weeks old). All behavioral tests used mature adult mice (16–20 weeks old) and were carried out during the light phase, the second part of the day. Both male and female mice were used in roughly equal numbers in all experiments. The following mouse lines were used: Vglut2-Cre (Cre expressed in all RGCs: JAX# 016963), Kcng4-Cre (Cre expressed in alpha RGCs: Jax# 029414), Cart-Cre (Cre expressed in ooDS-RGCs; JAX# 028533), and Opn4-Cre (Cre expressed in ipRGCs; Jax# 035925). To visualize RGCs labeled in each line, Vglut2-Cre, Cart-Cre, and Opn4-Cre lines were crossed to the reporter line Ai14 (JAX# 007914), which expresses Cre-dependent tdTomato. The Kcng4-Cre mouse line was crossed to reporter lines TSY1 or TSY15 (55), which express Cre-dependent YFP.

### Construction and generation of AAVs

Generation of AAV-FLEX-DTA has been previously described (56). To generate FLEX-YFP or FLEX-mCherry, a full sequence of YFP or mCherry was amplified by adding KpnI and NheI restriction sites at each end and subcloned into the AAV vector [pAAV-CAG-Flex-NheI-(gene of interest)-KpnI-WPRE-SV40 pA] (56). The plasmid carrying YFP and mCherry was previously described (57).

AAVs were produced using a triple-transfection, helper-free method, and purified as described (56). For transfection, a plasmid carrying the gene of interest, capsid 2/2 serotype packaging plasmid and delta F6 plasmid were used. Viral titers were determined by quantitative PCR using primers that recognize WPRE (5′-CGCTATGTGGATACGCTGCT-3′ and 5′- GCAAACACAGTGCACACCAC-3′); concentrated titers were >10^13^ viral genome particles/ml in all preparations. Viral stocks were stored at −80°C.

### Intraocular injection

Animals were treated with buprenorphine (0.05–0.1 mg/kg of body weight) before surgery and then anesthetized with a mixture of 100 mg ketamine plus 10 mg xylazine/kg of bodyweight. All drugs were injected intraperitoneally. Before injecting AAVs, a small hole was made on both eyes with an insect pin (size 00) to release intraocular pressure. AAV (0.5–1 µl of 1.5 × 10^13^ genome particles/ml) was delivered through the same hole by a pressure injector (Harvard Apparatus, PLI-100) connected to the back of an injection pipette. The pipette was advanced towards the ventral retina using a dissection microscope. Intraperitoneal injections of meloxicam (1–5 mg/kg of bodyweight) were given for 48 h after surgery. The injection location was confirmed by the ablation of Cre-expressing RGCs or by the presence of YFP or mCherry expression in the ventral retina. The virus typically spread beyond the ventral retina, extending into the dorsal retina but with more limited infection.

### Looming stimulus assay

Both male and female mice (16–20 weeks old) were utilized; the subject numbers were sex-adjusted. Behavioral tests were performed and analyzed 4 to 6 weeks after the AAV injections in a double-blind fashion.

The looming stimulus assay was conducted to assess visually triggered defensive responses as previously described (3). In brief, the testing apparatus contained a 40 cm × 40 cm × 30 cm (length × width × depth) open field arena enclosure of Plexiglas sidewalls (coated with a matte finish). For the freezing response, mice were evaluated in the absence of a shelter. For the escaping response, mice were evaluated in the presence of a shelter, which was placed in the corner of the testing arena. A computer monitor was mounted overhead to present the looming stimulus during testing. Mice were acclimated to the testing room for ∼45 minutes over the course of 3 days prior to the behavioral experiments.

The looming stimulus assay included acclimating each mouse to the open field arena (free exploration) for 10 min. Following the acclimation, the looming stimulus was delivered when the mouse was located within the stimulus area. Against a gray background, the looming stimulus was an expanding black disk that enlarged from 2^°^ to 20^°^ visual angle over 0.25 s and maintained its largest size for an additional 0.25 s. The stimulus subsequently disappeared for 0.5 s before repeating for a total of 10 iterations (1 stimulus per second for a total duration of 10 s). The looming stimulus was generated in MATLAB. Mouse behavior was recorded by a camera mounted on top of the testing arena and the recordings were stored through Spinnaker video capture software.

The mouse freezing response was defined as complete immobility (including no movement of the head and tail) except for breathing and measured over 2 minutes post-stimulus onset. Of note, two animals (one control mouse in each of the Kcng4-Cre and Cart-Cre groups) maintained freezing until the end of recording. The escaping response was defined as a combination of freezing and fleeing behavior, with fleeing defined as rapid movement towards the shelter. All behaviors video-recorded during the looming stimulus assay were analyzed using BORIS 8.21, an open-source tagging software program (58). For escaping behavior, mouse movement was also traced and analyzed with the MTrackJ plugin in ImageJ software (59). Once the mouse entered the shelter after stimulus onset, tracing was stopped. The location of the mouse's nose was manually tracked for each video frame (0.02 s per frame) 10 s before and after stimulus onset (or until the mouse entered the shelter). Manual tracking of nose movement generated X and Y coordinates as a function of time. The instantaneous speed of each mouse movement was quantified by dividing X and Y coordinate differences by the time differences of two consecutive frames. The average speed was then calculated by averaging the instantaneous speeds.

To estimate how broadly the retina should be infected by AAVs in order to alter behavior, two criteria were considered: (i) Given that the maximum size of the looming stimulus is 20 degrees, virus infections within the ventral retina should cover at least ∼0.28 mm^2^ of retinal area (∼0.60 mm diameter), corresponding to 20 degrees of stimulus size; (ii) Correlation analysis between the extent of the infected area and behavioral changes using the Vglut2-Cre line revealed an exponential relationship [fitted line: *y* = 24.2 exp(-*x*/0.185)]. Application of three-sigma limits using the space constant of 0.185 showed that at least ∼0.56 mm^2^ of retinal area (∼0.84 mm diameter) should be infected. Together, we used >0.9 mm^2^ of ventral retina (∼1.07 mm diameter) as a conservative threshold for valid testing of an RGC type's role in defensive behavior.

### Light/dark exploration test

The light/dark exploration test was performed 3–5 days after the looming stimulus assay to evaluate an animal's general visual ability as previously described (48–50). In brief, the 40 × 40 × 30 cm (length × width × depth) testing arena was evenly divided into a transparent Plexiglas chamber and a black opaque Plexiglas chamber. The dark compartment had a removable black Plexiglas lid and a small entryway. Prior to the experiments, mice were acclimated to the testing room for ∼45 minutes. Experiments were conducted in a room with the overhead light off, and a bright 120 W lamp directed at the transparent compartment of the testing area. A mouse was placed in the black Plexiglass box, and its behavior (free exploration of the arena) was video recorded for 10 min. The recordings were analyzed for the total time spent in the brightly lit compartment of the arena using BORIS 8.21.

### Immunohistochemistry

Adult mice were sacrificed after all behavioral tests. Mice were anesthetized with an intraperitoneal injection of 100 mg ketamine/10 mg xylazine per kg of bodyweight and perfused transcardially with 4% Paraformaldehyde (PFA) in 0.1 M Phosphate-Buffered Saline (PBS). Following perfusion, retinas were dissected and fixed in 4% PFA/PBS for 1 h at 4°C. For immunostaining, retinas were then washed twice (5 minutes each) with PBS, blocked for 1 h in 3% donkey serum/0.1% Triton X-100/PBS and rat anti-mouse CD16/CD32 (mouse Fc Block;1:500, BD Biosciences, 553141) at room temperature, and incubated with primary antibodies for 3 days at 4°C and subsequently with secondary antibodies for 2 h at room temperature.

The following primary antibodies were used: chicken anti-GFP (1:3,000, Aves Laboratories, GFP-1020), rat anti-mCherry (1:2,000, Kerafast, EST202), rabbit anti-S opsin (1:2,000, Millipore, AB5407), rabbit anti-DsRed (1:3,000, Clontech, 632496), rabbit anti-Cart (1:1000, Phoenix Pharmaceuticals, H-003-62), goat anti-Brn3a (1:3,000, Santa Cruz, sc-31984), goat anti-S opsin (1:200, Santa Cruz, sc-14365). Secondary antibodies were conjugated to Alexa Fluor-488, Cy3, Cy5 and diluted at 1:500 (Jackson ImmunoResearch Laboratories, 703-545-155, 711-545-152, 711-165-152, 705-175-147, 705-165-147, 712-165-153, or Thermo Fisher, A-11055).

### Image acquisition and analysis

Images of the ventral whole-mount retina (identified by S-opsin staining) were taken on a Zeiss Imager M2 fluorescence microscope and a Zeiss LSM 800 confocal microscope. Z-stacks were collected with 1 µm steps using a 20 × objective (NA 0.8) or with 4 µm steps using a 10 × objective (NA 0.45). Images were analyzed using ImageJ software (National Institutes of Health, Bethesda, MD).

For cell counting, confocal images were cropped to 300 µm × 300 µm areas halfway between the optic nerve and the ventral edge of the retina. For quantification, two ventral areas of each retina were analyzed, and total cell counts of these areas were averaged between left and right retinas. For Vglut2-Cre:: Ai14 control mice, total counts of cells that express both YFP and tdTomato were collected, averaged, and presented as a percentage of co-expression level. For Kcng4-Cre:: TSY1 (or TSY15), Cart-Cre:: Ai14, and Opn4-Cre:: Ai14 mice, total counts of cells that express Brn3a were collected, averaged, and compared between control and DTA groups. For each of the more selective Cre lines, only a fraction of RGCs should be ablated in the DTA-infected area, and thus we expected the number of Brn3a cells to be only partially reduced (by ∼10–20%).

For measuring DTA-infected area, images of the ventral whole-mount retina were taken. Using the ZEN 2012 (Blue edition) software or Image J, all ventral areas with 90–100% of RGCs ablated were manually outlined with the contour graphics tool and measured. Area measurements were correlated with freezing times to determine minimum infection area necessary to produce significant behavioral changes.

### Quantification and statistical analysis

Cell counting was conducted in two ventral areas of each retina per animal in six animals per condition. Behavioral data were analyzed between nine and twenty-three animals. Animals of either sex were utilized for analysis.

All data were analyzed with GraphPad Prism software (GraphPad Software; Dotmatics, San Diego, CA) and reported as mean ± SEM. Unpaired two-tailed Student's t-test was conducted for all two-group comparisons. Two-way ANOVA with subsequent Tukey post-hoc test was utilized for multiple group comparisons. Statistical significance was defined as *P* < 0.05. Further details on statistical tests, exact values of *n*, mean ± SEM, and statistical significance for each data set are outlined either in figure legends or Tables S1 and S2. Statistical significance was determined when *P* < 0.05 (*), *P* < 0.01 (**), *P* < 0.001 (***), *P* < 0.0001 (****).

## Supplementary Material

pgae423_Supplementary_Data

## Data Availability

The data supporting the findings of this article are available within the article and/or its Supplementary Materials.
