# Supplementary material for: Specific retinal neurons regulate context-dependent defensive responses to visual threat
Source: PNAS Nexus. 2024 Sep 24;3(10):pgae423. doi: 10.1093/pnasnexus/pgae423 (PMC11443969; doi:10.1093/pnasnexus/pgae423)
Supplement: pgae423_Supplementary_Data [file pgae423_supplementary_data.pdf]

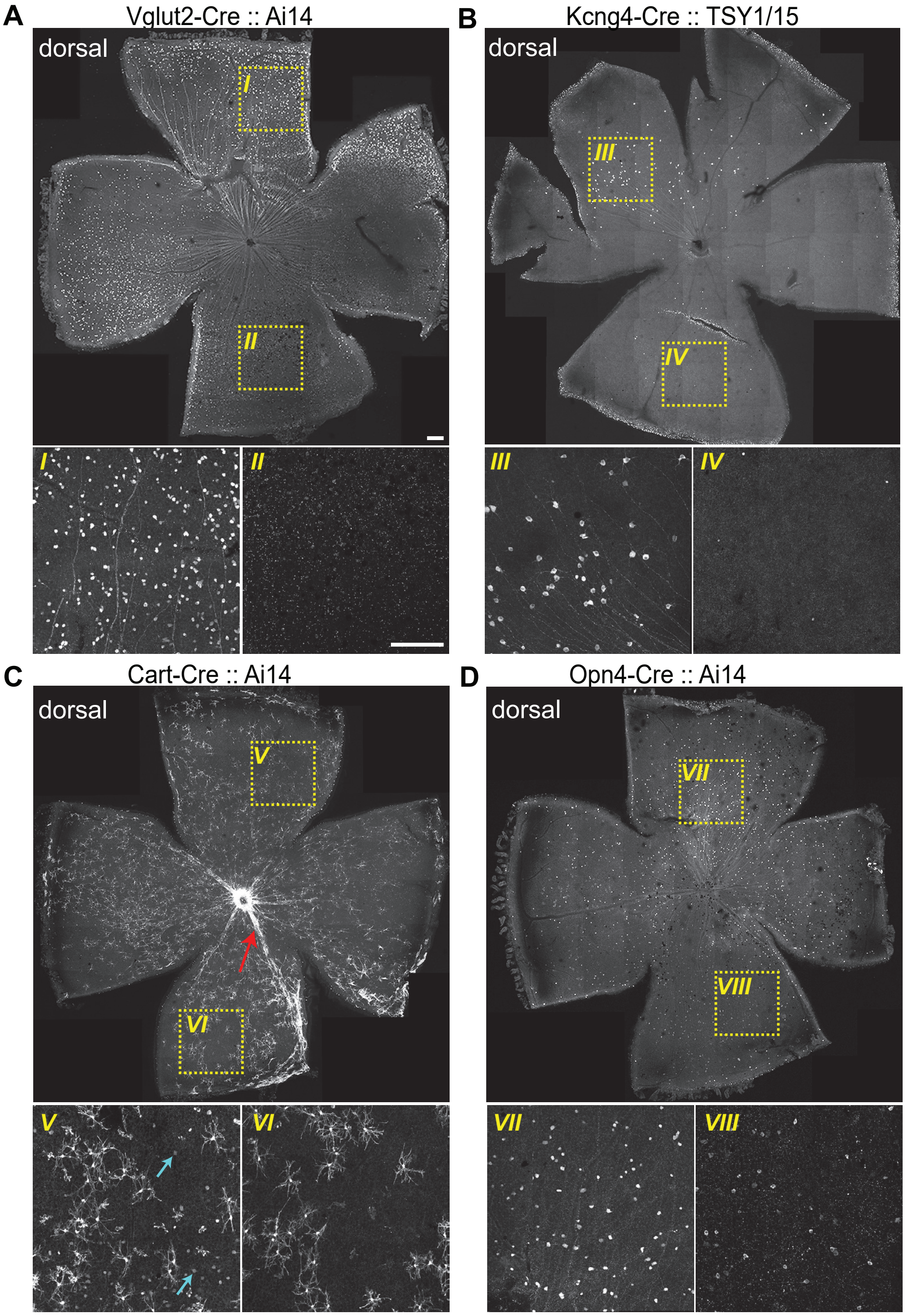

Fig. S1: Assessment of ablated retinal area in each transgenic line (related to Figs. 1-4) AAV-FLEX-DTA was injected into both eyes of mice from each transgenic line crossed to a reporter line. Following behavioral analysis, retinas were dissected and evaluated for the DTA-infected area using the loss of reporter expression. Vglut2 -Cre :: Ai14 (**A**), Kcng4-Cre :: TSY1/15 (**B**), Cart-Cre :: Ai14 (**C**) and Opn4-Cre :: Ai14 (**D**). Magnified view of the boxed areas (***I* - *VIII***) showing a clear loss of RGCs in the ventral retinas. A red arrow (***C***) indicates a cluster of uncharacterized cells in the inner limiting membrane of Cart-Cre :: Ai14 retinas. These cells (presumed astrocytes) have multiple thick processes, making them easily distinguishable from labeled RGCs (blue arrows). Scale bars: 200 µm.

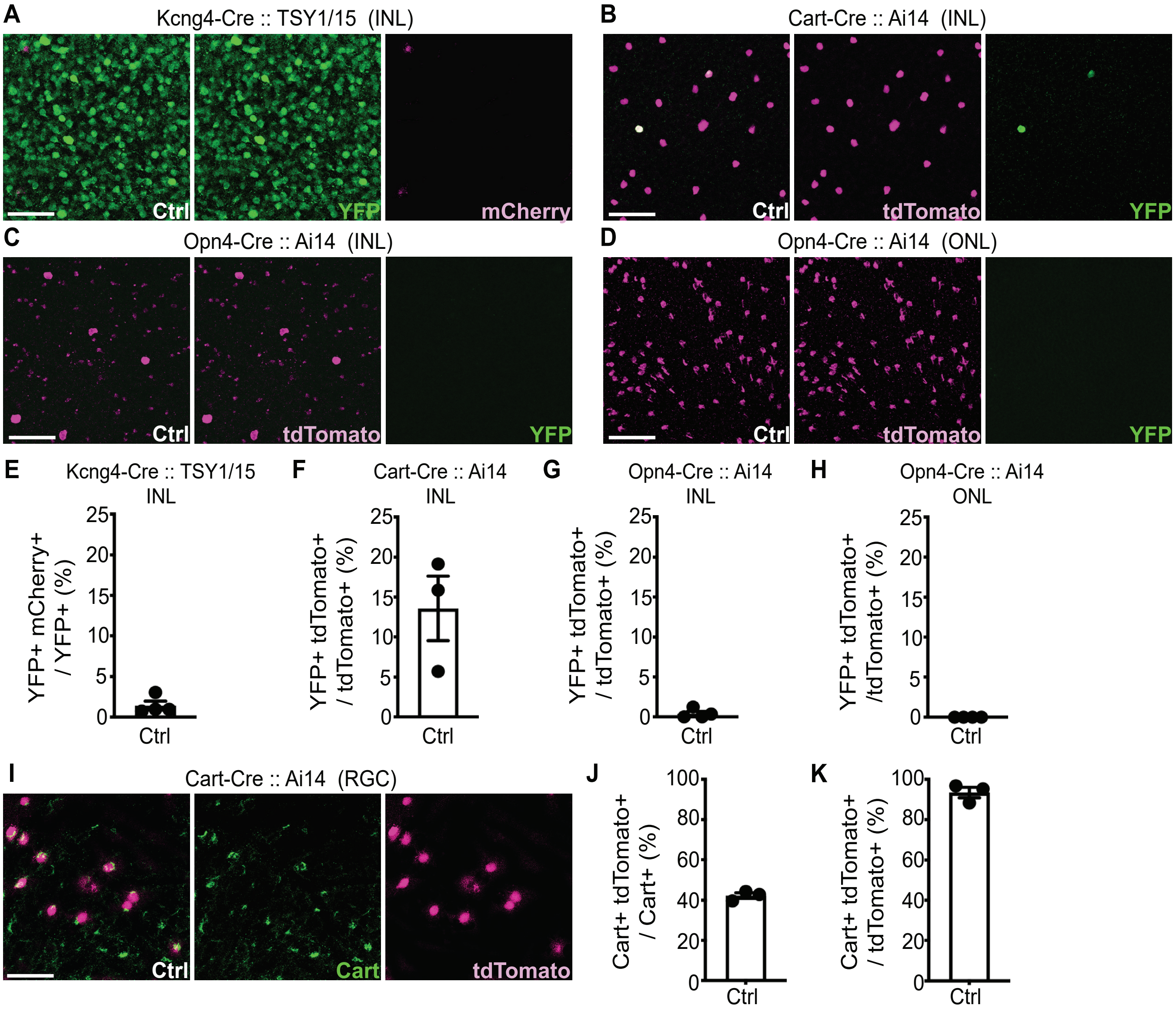

Fig. S2: Histological analysis of each transgenic line (related to Figs. 2-4)
A-H. AAV-FLEX-mCherry (or YFP) was injected into both eyes of each transgenic line crossed to a reporter line. Following behavioral analysis, retinas were immunostained against GFP (green), DsRed (magenta), S-opsin and Brn3a to examine the retinal ganglion cell (RGC) layer. We also examined the inner and outer nuclear layers (INL and ONL) and found that the AAV2/2 serotype used here barely infected neurons of Kcng4-Cre :: TSY1 (TSY15) in the INL (**A, E**), Cart-Cre :: Ai14 in the INL (**B, F**), Opn4-Cre :: Ai14 in the INL (**C, G**) and Opn4-Cre :: Ai14 in the ONL (**D, H**). No labeled neurons were found in the ONL of Kcng4-Cre :: TSY1 (TSY15) and Cart-Cre :: Ai14. **I-K.** To examine the number of ooDS-RGCs labeled in Cart-Cre :: Ai14, retinas were dissected, immunostained against Cart (green) and DsRed (magenta). Quantification revealed that ~42.0% of Cart+ cells were td-Tomato+, similar with reported values (34), and ~94.2% of td-Tomato+ cells were Cart+. Scale bars: 50 μm.

Table S1: Numerical values of histological analysis in each transgenic line. Related to Figures 1-4.

Retinal Ganglion Cell (RGC) Layer

	**Vglut2-Cre :: Ai14**			**Cart-Cre :: Ai14**			
	Sample Size	YFP+ (cells/mm^2)	YFP+ tdTomato+ (cells/mm^2)	Sample Size	Cart+ (cells/mm^2)	tdTomato+ (cells/mm^2)	Cart+ tdTomato+ (cells/mm^2)
**Control**	5	2636 ± 250	2509 ± 263	3	576 ± 84	257 ± 24	242 ± 28

	**Kcng4-Cre :: TSY1/15**		**Cart-Cre :: Ai14**		**Opn4-Cre :: Ai14**	
	Sample Size	Brn3a+ (cells/mm^2)	Sample Size	Brn3a+ (cells/mm^2)	Sample Size	Brn3a+ (cells/mm^2)
**Control**	6	3964 ± 152	6	3730 ± 165	6	3788 ± 110
**DTA**	6	3194 ± 181	6	3044 ± 236	6	3238 ± 124

	**Opn4-Cre :: Ai14**	
	Sample Size	M4+ (cells/mm^2)
**Control**	4	77.8 ± 2.3
**DTA**	4	8.3 ± 2.8

Inner Nuclear Layer (INL) & Outer Nuclear Layer (ONL)

	**Kcng4-Cre :: TSY1/15 (INL)**			**Cart-Cre :: Ai14 (INL)**		
	Sample Size	YFP+ (cells/mm^2)	YFP+ Cherry+ (cells/mm^2)	Sample Size	tdTomato+ (cells/mm^2)	YFP+ tdTomato+ (cells/mm^2)
**Control**	4	9939 ± 1319	150 ± 65	3	602 ± 52	81 ± 24

	**Opn4-Cre :: Ai14 (INL)**			**Opn4-Cre :: Ai14 (ONL)**		
	Sample Size	tdTomato+ (cells/mm^2)	YFP+ tdTomato+ (cells/mm^2)	Sample Size	tdTomato+ (cells/mm^2)	YFP+ tdTomato+ (cells/mm^2)
**Control**	4	2674 ± 441	14 ± 11	4	3967 ± 314	0 ± 0

Table S2: Numerical values of behavior analysis in each transgenic line. Related to Figures 1-4.

Vglut2-Cre :: Ai14						
**Without Shelter**	**Looming**					**Light/Dark**
	Sample Size	Freezing Time (s)	Escape Latency (s)	Average Speed (cm/s)		Time in the Light (s)
				Before Stimulus	After Stimulus	
**Control$_{Total}$**	9	34.0 ± 9.0	-	-	-	187.9 ± 18.1
**DTA$_{Total}$**	23	10.1 ± 2.8	-	-	-	207.1 ± 13.5
**DTA$_{Total_0.9mm2}$**	13	2.4 ± 1.5	-	-	-	236.7 ± 16.8
Kcng4-Cre :: TSY1/15						
**Without Shelter**	**Looming**					**Light/Dark**
	Sample Size	Freezing Time (s)	Escape Latency (s)	Average Speed (cm/s)		Time in the Light (s)
				Before Stimulus	After Stimulus	
**Control$_{Total}$**	13	33.3 ± 10.1	-	-	-	201.2 ± 13.8
**DTA$_{Total}$**	12	19.1 ± 6.6	-	-	-	190.9 ± 15.4
**DTA$_{Total_0.9mm2}$**	9	24.0 ± 8.3	-	-	-	201.0 ± 17.8
**With Shelter**	**Looming**					**Light/Dark**
	Sample Size	Freezing Time (s)	Escape Latency (s)	Average Speed (cm/s)		Time in the Light (s)
				Before Stimulus	After Stimulus	
**Control$_{Total}$**	12	1.5 ± 0.8	-	-	-	186.1 ± 18.6
**DTA$_{Total}$**	15	7.9 ± 2.8	-	-	-	191.6 ± 10.1
**DTA$_{Total_0.9mm2}$**	11	7.4 ± 3.2	-	-	-	205.4 ± 10.4
**Control$_{Escaping}$**	11	-	1.6 ± 0.6	10.0 ± 0.9	42.4 ± 8.6	187.7 ± 20.3
**DTA$_{Escaping}$**	9	-	4.3 ± 0.7	10.6 ± 1.3	12.6 ± 2.5	199.2 ± 12.9
**DTA$_{Escaping_0.9mm2}$**	7	-	4.1 ± 0.9	10.8 ± 1.6	13.9 ± 3.1	209.4 ± 13.8
Cart-Cre :: Ai14						
**Without Shelter**	**Looming**					**Light/Dark**
	Sample Size	Freezing Time (s)	Escape Latency (s)	Average Speed (cm/s)		Time in the Light (s)
				Before Stimulus	After Stimulus	
**Control$_{Total}$**	11	32.8 ± 10.5	-	-	-	176.9 ± 21.9
**DTA$_{Total}$**	9	21.3 ± 2.5	-	-	-	175.9 ± 19.8
**DTA$_{Total_0.9mm2}$**	8	20.7 ± 2.8	-	-	-	172.0 ± 22.0
**With Shelter**	**Looming**					**Light/Dark**
	Sample Size	Freezing Time (s)	Escape Latency (s)	Average Speed (cm/s)		Time in the Light (s)
				Before Stimulus	After Stimulus	
**Control$_{Total}$**	12	4.9 ± 3.0	-	-	-	182.8 ± 13.0
**DTA$_{Total}$**	14	3.7 ± 1.9	-	-	-	192.6 ± 15.4
**DTA$_{Total_0.9mm2}$**	14	3.7 ± 1.9	-	-	-	192.6 ± 15.4
**Control$_{Escaping}$**	10	-	2.2 ± 0.7	9.1 ± 0.8	39.7 ± 7.0	181.5 ± 14.2
**DTA$_{Escaping}$**	11	-	1.5 ± 0.6	10.9 ± 0.8	46.1 ± 5.6	183.7 ± 18.2
**DTA$_{Escaping_0.9mm2}$**	11	-	1.5 ± 0.6	10.9 ± 0.8	46.1 ± 5.6	183.7 ± 18.2
Opn4-Cre :: Ai14						
**Without Shelter**	**Looming**					**Light/Dark**
	Sample Size	Freezing Time (s)	Escape Latency (s)	Average Speed (cm/s)		Time in the Light (s)
				Before Stimulus	After Stimulus	
**Control$_{Total}$**	15	45.8 ± 7.8	-	-	-	152.4 ± 17.6
**DTA$_{Total}$**	10	20.7 ± 5.2	-	-	-	152.6 ± 13.8
**DTA$_{Total_0.9mm2}$**	6	11.9 ± 3.5	-	-	-	145.9 ± 19.3
**With Shelter**	**Looming**					**Light/Dark**
	Sample Size	Freezing Time (s)	Escape Latency (s)	Average Speed (cm/s)		Time in the Light (s)
				Before Stimulus	After Stimulus	
**Control$_{Total}$**	12	9.1 ± 3.3	-	-	-	173.4 ± 13.1
**DTA$_{Total}$**	10	4.7 ± 1.8	-	-	-	200.8 ± 18.1
**DTA$_{Total_0.9mm2}$**	8	5.3 ± 2.2	-	-	-	206.4 ± 20.3
**Control$_{Escaping}$**	4	-	-	-	-	-
**DTA$_{Escaping}$**	3	-	-	-	-	-
**DTA$_{Escaping_0.9mm2}$**	2	-	-	-	-	-
